# 843. Targeted HIV Testing for Patients Hospitalized for COVID-19

**DOI:** 10.1093/ofid/ofab466.1038

**Published:** 2021-12-04

**Authors:** Michael D Virata, Merceditas Villanueva, Janet Miceli

**Affiliations:** Yale University School of Medicine, New Haven, CT

## Abstract

**Background:**

SARS-CoV-2 causes a severe respiratory illness known as COVID-19. Treatment options in the early portion of the COVID-19 pandemic included the use of antiretroviral agents i.e. protease inhibitors (PIs) such as lopinavir (LPV) that had been shown to have activity against the main proteases of SARS-CoV-2 in vitro but with very limited clinical data. Prior to the use of PIs, HIV testing would be indicated to ensure that patients who were not previously diagnosed with HIV would start appropriate HIV treatment. In this unique situation, HIV testing would be utilized not based on traditional HIV risk factors.

**Methods:**

We performed a retrospective search from a specific systems database of patients admitted to Yale-New Haven Health System (YNHHS) with a diagnosis of COVID-19 infection. We identified a subset of patients who were HIV tested. Most were done prior to initiating PI treatment. Demographics, comorbidity scores and specific underlying conditions were also tabulated. We performed Kruskal Wallis and Chi-Squared analysis to test for significance between HIV- and HIV+ patients.

**Results:**

The total no. of patients admitted to the YNHHS with COVID-19 infection between the period from January 6, 2020 to January 6, 2021 was 5776. A cohort 964 (16.7%) patients were screened for HIV. Much of the testing occurred in the early COVID periods (Figure 1) when PIs were considered as part of the treatment algorithm. Sixty-seven (0.07%) patients tested HIV+ with 3 (0.003%) being newly diagnosed (Fig 2). Compared to HIV- patients, HIV+ were more likely to be identified as Black, with higher mean Elixhauser Comorbidity scores and significant associations with conditions such as hypertension, pulmonary disease, complicated diabetes, liver disease, renal failure and depression (Table 1). These co-morbidities have been correlated with higher risk of hospitalization for people living with HIV (PWH).

Figure 2. COVID Admission and HIV Status

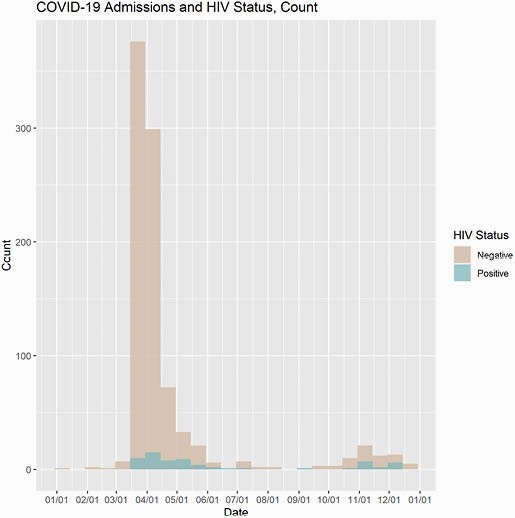

The graph represents HIV testing results over the entire study period.

Table 1. Demographics and Comorbidites

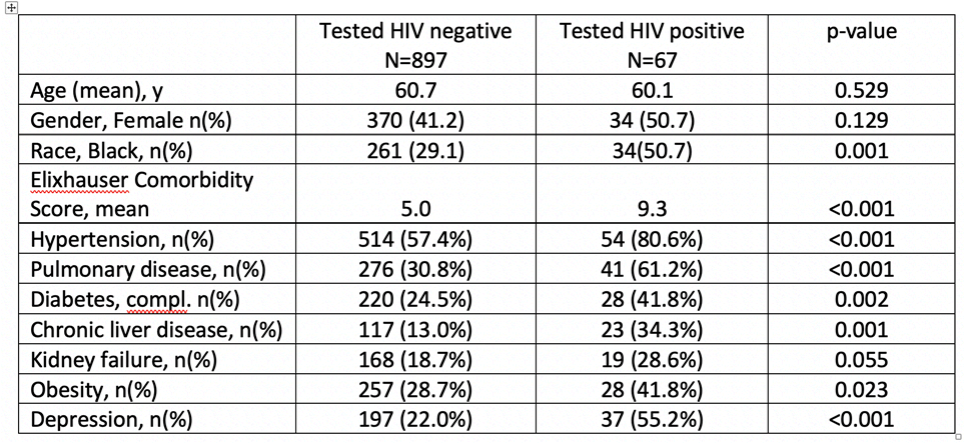

Represents demographics and comorbidities of HIV- & HIV+ patients

Figure 1. COVID Admissions and HIV Testing

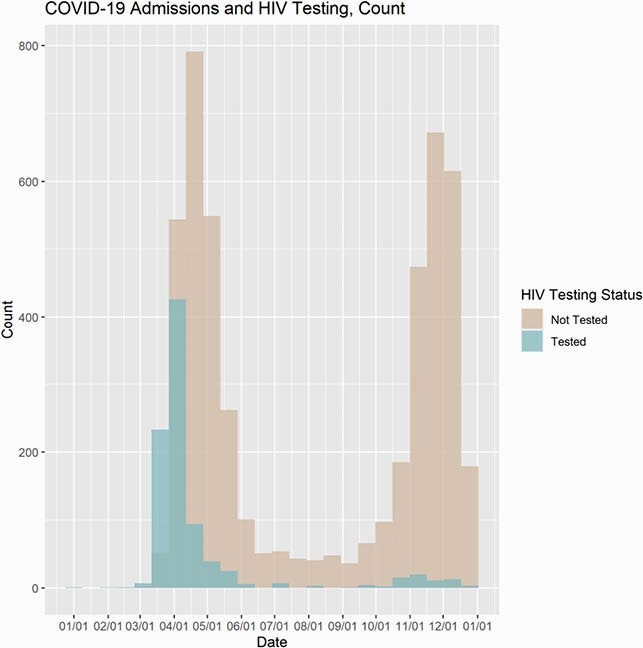

COVID admissions over time and the performance of HIV testing

**Conclusion:**

This is one of the first reports on targeted HIV testing for patients not using identifiable traditional HIV risk factors who were admitted to a large healthcare system for COVID19 infections. The percentage of newly HIV diagnosed patients from this cohort was considered to be < known HIV infection rates for our population. The majority of PWH were already established in care prior to their COVID19 diagnosis.

**Disclosures:**

**All Authors**: No reported disclosures

